# Persistent low-density infection in areas at risk of malaria reintroduction in Malaysia

**DOI:** 10.1371/journal.pone.0329219

**Published:** 2025-11-20

**Authors:** Adela Ida Jiram, Shamilah Hisam, Mohd Hafizi Abdul Hamid, Jenarun Jelip, Rahmah Noordin, Kamarul Imran Musa, Nurhainis Ogu Salim, Noor Azian Md Yusuf, Nur Fariha Amir, Aliaa Rasyidina, Nor Parina Ismail, Dg Izyan Hazwani Aziz, Nur Hafizah Abdullah, Aqidah Kep, Zaini Hussin, Aziah Ismail

**Affiliations:** 1 Parasitology Unit, Infectious Disease Research Centre, Institute for Medical Research, Shah Alam, Selangor, Malaysia; 2 Institute for Research in Molecular Medicine (INFORMM), Health Campus, Universiti Sains Malaysia, Kubang Kerian, Kelantan, Malaysia; 3 Disease Control Division, Ministry of Health Malaysia, Putrajaya, Malaysia; 4 Department of Public Health Medicine, Faculty of Medicine and Health Sciences, University Malaysia Sabah, Kota Kinabalu, Sabah, Malaysia; 5 Department of Parasitology and Medical Entomology, Faculty of Medicine, Universiti Kebangsaan Malaysia, Cheras, Kuala Lumpur, Malaysia; 6 Department of Community Medicine, School of Medical Sciences, Universiti Sains Malaysia, Kubang Kerian, Kelantan, Malaysia; 7 Kelantan State Health Department, Wisma Persekutuan, Kota Bharu, Kelantan, Malaysia; Menzies School of Health Research, AUSTRALIA

## Abstract

Malaysia successfully achieved zero indigenous human malaria cases since 2018. However, challenges persist from *Plasmodium knowlesi* (zoonotic malaria) and low-density infections, posing reintroduction risks in previously malaria-free areas. Addressing these hidden infections is critical for sustaining Malaysia’s elimination gains. This study investigated the persistence of low-density malaria transmission in high-risk localities declared malaria-free for at least three consecutive years. A community-based cross-sectional survey was conducted from June to October 2020 in 23 high-receptivity localities across Sabah, Perak, Kelantan, and Johor. Blood samples from asymptomatic residents were screened via conventional microscopy and nested PCR (nPCR) targeting the *Plasmodium* 18S rRNA gene, with positive nPCR products species-determined. Sociodemographic and geospatial data were analyzed for associations with infection status. Of 3,322 asymptomatic individuals, no infections were detected by microscopy, whereas nPCR revealed a low-density malaria prevalence of 1.86% (62/3,322). Infections comprised *P. malariae* (40.3%), *P. vivax* (29.0%), *P. knowlesi* (24.2%), *P. falciparum* (1.6%), *P. cynomolgi* (1.6%), and mixed *P. vivax/P. knowlesi* (3.2%). All PCR-positive cases originated from Sabah and an Orang Asli settlement in Perak. Adults (≥17 years) constituted the majority (~68%), with no significant difference in prevalence by gender or previous malaria history (p > 0.05). Asymptomatic low-density malaria infections persist in purportedly malaria-free communities, remaining undetectable by routine microscopy. These hidden parasite reservoirs pose a risk for malaria reintroduction, especially in receptive areas. Malaria surveillance programs must thus incorporate highly sensitive diagnostic tools to detect low-density infections and safeguard elimination gains. Intensified, targeted interventions in identified “malaria hotspots”, including community engagement and vector control, are crucial to eliminate residual foci and prevent disease resurgence.

## Introduction

Malaria remains a significant global health challenge, affecting millions of people annually and impeding socioeconomic development in endemic regions [1]. Recognizing the need for intensified elimination strategies, the World Health Organization (WHO) launched the Elimination-2020 (E-2020) initiative in 2017 to support countries in accelerating malaria elimination [2]. Malaysia was identified as one of 21 countries with the potential to reach zero indigenous malaria cases by 2020, and it has since exemplified progress by interrupting human malaria transmission nationwide. However, Malaysia’s success is tempered by the rise of zoonotic malaria. *Plasmodium knowlesi*, a simian malaria parasite that infects humans, has become the predominant cause of malaria in the country, accounting for the majority of cases in recent years [3,4]. This zoonotic reservoir cannot be eliminated through traditional human-focused measures and continues to threaten the forested regions.

Compounding this challenge is the issue of low-density malaria—infectious cases with parasite densities below the detection threshold of conventional microscopy [5–8]. Such low-level infections, often without symptoms, serve as hidden reservoirs that can perpetuate transmission and undermine elimination efforts [9,10]. In Malaysia’s context, these “invisible” infections could allow malaria to persist or re-emerge even after apparent interruption of transmission. Sustaining the nation’s gains in achieving zero indigenous malaria will require addressing this hidden burden head-on [11].

Despite robust surveillance and control programs, low-density malaria infection remains understudied in Malaysia, particularly in areas deemed at risk of reintroduction (high receptivity and vulnerability) [12,13]. Traditional diagnostics (microscopy and rapid diagnostic tests) fail to detect low-density parasitaemia, leaving a portion of infections undiagnosed and untreated [14–17]. It creates a gap in our understanding of residual malaria transmission dynamics in elimination-phase settings. Therefore, this study aimed to investigate the epidemiology of low-density malaria in asymptomatic individuals in high-risk, officially malaria-free localities in Malaysia using sensitive molecular diagnostics, to detect lingering *Plasmodium* infections and inform evidence-based elimination strategies.

## Materials and methods

### Study design and setting

A community-based cross-sectional study was conducted from June 24 to October 31 2020 in 23 localities across seven districts of Malaysia that were identified as having a high risk for malaria reintroduction. These study sites were located in the states of Sabah (Kota Marudu and Keningau districts), Perak (Hulu Perak district, specifically the RPS Pos Kemar resettlement), Johor (Mersing and Segamat districts), and Kelantan (Gua Musang and Kuala Krai districts). Each locality had been declared malaria-free (zero indigenous cases) for at least three consecutive years before the study yet remained highly receptive and vulnerable to malaria due to ecological and demographic factors (e.g., presence of mosquito vectors, proximity to forest or international borders and mobile populations). The local communities included rural indigenous groups in Sabah, an Orang Asli (aboriginal) population in Hulu Perak and rural villages in Johor and Kelantan. These communities typically engage in agriculture, small-scale farming, or forest-related activities which exposes them to malaria vectors.

### Ethical statement

This study was registered with the National Medical Research Register (NMRR-19-470-47341) and received ethical approval from the Medical Research and Ethics Committee (MREC), Ministry of Health Malaysia (Ref: KKM/NIHSEC/P19-884). Additional support and permissions were obtained from the respective State Health Departments. Participation was voluntary, and written informed consent was obtained from all adult participants. For any adult participants who were not literate, informed consent was obtained via thumb-printing, witnessed by an independent third party, as approved by the Medical Research and Ethics Committee (MREC). For minors, assent was obtained along with parental or guardian consent. All personal data were anonymized and handled confidentially. Individuals who tested positive by PCR were subsequently followed up by local health authorities and treated in accordance with national malaria treatment guidelines [2,18]. Participants who became microscopy-positive were managed as clinical malaria cases, receiving appropriate treatment per the national protocol.

### Sample size and sampling strategy

The sample size calculation was based on an anticipated prevalence of low-density malaria of 1.7%, as reported in a previous related study conducted in Malaysia [16]. We aimed for 95% confidence and ≥90% power to detect infections [19]. To account for anticipated non-response rates (due to logistical challenges, difficult terrain, participant mobility, and community reluctance), the sample size was conservatively inflated by 30% [11,20,21]. Thus, approximately 3,500 individuals were targeted for sampling across all sites. These targets were allocated to each state proportional to population size and risk level, for example, larger samples in high-risk localities like Pos Kemar in Perak and Kota Marudu in Sabah and smaller samples in lower-population villages. Stratified random sampling was employed: households were randomly chosen from community lists within each selected locality, and all consenting resident members of each selected household were included. Participants of all ages were enrolled after obtaining written informed consent (and assent for minors). Inclusion criteria for participants were: 1) long-term residents of the study area (defined as residing for at least six months prior to the study) to ensure representation of the local population and capture ongoing transmission dynamics; and 2) voluntary provision of written informed consent (and assent for minors). Exclusion criteria included: 1) individuals presenting with clinical malaria symptoms at the time of sampling; and 2) individuals who did not provide consent or assent.

### Baseline demographics and study questionnaire

Baseline socio-demographic data were collected from all participants using a structured questionnaire administered by trained field staff. Data collected included gender, age, religion, ethnicity, educational level and occupation. Geographic information, including village Global Positioning System (GPS) coordinates, was recorded to map the spatial distribution of participants. However, participants were not asked about their knowledge of malaria, such as transmission routes, prevention methods, or previous experiences with malaria infections or antimalarial medication use. Symptoms related to malaria, including fever, vomiting, convulsions, headache, loss of appetite, and general body malaise, were documented based on participants’ recall rather than clinical assessment. Fever was defined as an axillary body temperature of ≥37.5°C, measured using digital thermometers (Omron, Turkey). Participants were asked if they had experienced fever or received antimalarial treatment within 14 days before enrolment.

### Blood sample collection and preparation

Blood samples were collected via venepuncture from all consenting individuals residing in the same locality, which shared similar ecological conditions. A volume of 5 ml was obtained from adults and older children [22], 3 ml from children aged 3–5 years, and 1–2 ml from younger children [23]. Samples were transferred into anticoagulant-containing vacuum tubes: either PAXgene® Blood DNA Tubes or BD Vacutainer® EDTA tubes [22]. PAXgene® tubes were preferred in this study due to their ability to stabilize genomic DNA at room temperature for up to 14 days. These tubes contain chemical additives that lyse cells and precipitate DNA, thereby preventing degradation [24]. However, when the minimum required volume of 2.5 ml could not be obtained (particularly from younger children), BD Vacutainer® tubes containing EDTA were used instead. These EDTA tubes were stored in a cool box and temporarily kept at −20°C at the nearest local clinic before being transported to the laboratory.

For participants who declined venepuncture, blood samples were obtained via finger prick. Approximately 0.5 ml of blood was spotted onto Whatman® 3MM filter paper, air-dried, and individually stored in zip-lock plastic bags with desiccants for later PCR analysis [25,26]. Additionally, a portion of the blood was directly utilized to prepare thick and thin blood smears for microscopic analysis [22,27].

### Microscopic examination

Thin and thick blood films were prepared in duplicate from each blood sample, stained with 3% Giemsa, and then examined under a light microscope by highly experienced microscopists at both clinical and research laboratories, as an internal quality control measure to ensure the reliability and accuracy of parasite detection and counting. Asexual parasites were counted against 200 leukocytes, while sexual parasites were counted against 500 leukocytes. If the asexual parasite counts were fewer than 10, the reading was extended to 500 leukocytes [27].

Two highly experienced microscopists examined each slide independently, and results with less than 50% difference between the two readings were considered definitive. A third blinded microscopist conducted an independent evaluation for slides with discordant results and the majority consensus was considered final. A slide was declared negative if no parasites were observed after scanning 200 high-power fields.

### Genomic DNA extraction and molecular detection of malaria parasites by PCR

Genomic DNA was extracted from whole blood collected in PAXgene® Blood DNA Tubes or BD Vacutainer® blood tubes and from bloodspots on filter paper, using commercially available kits. The PAXgene™ Blood DNA Kit (QIAGEN, Germany) was used for PAXgene® Blood DNA Tubes, while the QIAamp® DNA Mini Kit (QIAGEN, Germany) was employed for DNA extraction from BD Vacutainer® blood tubes and bloodspots on filter paper. The extracted DNA eluates were stored at −20°C until further analysis.

Nested PCR (nPCR) assays were conducted using genus- and species-specific primers targeting the small subunit ribosomal RNA (18S rRNA) genes to detect human (*Plasmodium falciparum*, *Plasmodium vivax*, *Plasmodium malariae*, *Plasmodium ovale*) [7,8,25] and simian (*P. knowlesi*, *P. inui*, *P. cynomolgi*, *P. fieldi*, *P. coatneyi*) [28] malaria species. Known positive and negative controls from previously diagnosed cases and uninfected individuals were included in each run. PCR primer sequences and annealing temperatures are provided in Table 1.

**Table 1:** List of primers including name, nucleotide sequence, PCR step, fragment size and specificity of the nested PCR.

*Plasmodium*	Primers	Sequence (5’ – 3’)	Annealing temperature (^o^C)
Genus-specific	rPLU1	TCAAAGATTAAGCCATGCAAGTGA	56
	rPLU5	CCTGTTGTTGCCTTAAACTTC	
Genus-specific	rPLU3	TTTTTATAAGGATAACTACGGAAAAGCTGT	59
	rPLU4	CCCGTCATAGCCATGTTAGGCCAATACC	
**Human malarias**			
*vivax*	rVIV1	CGCTTCTAGCTTAATCCACATAACTGATAC	60
	rVIV2	ACTTCCAAGCCGAAGCAAAGAAAGTCCTTA	
*falciparum*	rFAL1	TTAAACTGGTTTGGGAAAACCAAATATATT	
	rFAL2	ACACAATGAACTCAATCATGACTACCCGTC	
*ovale*	rOVA1	ATCTCTTTTGCTATTTTTTAGTATTGGAGA	
	rPLU2	ATCTAAGAATTTCACCTCTGACATCTG	
*malariae*	rMAL1	ATAACATAGTTGTACGTTAAGAATAACCCC	
	rMAL2	AAAATTCCCATGCATAAAAATTATACAAA	
*knowlesi*	Pmk8	GTTAGCGAGAGCCACAAAAAAGCGAAT	61
	Pmkr9	ACTCAAAGTAACAAAATCTTCCGTA	
**Simian malarias**			
*knowlesi*	Kn1f	CTCAACACGGGAAAACTCACTAGTTTA	62
	Kn3r	GTATTATTAGGTACAAGGTAGCAGTATGC	
*coatneyi*	PctF1	CGCTTTTAGCTTAAATCCACATAACAGAC	62
	PctR1	GAGTCCTAACCCCGAAGGGAAAGG	
*cynomolgi*	CY2F	GATTTGCTAAATTGCGGTCG	60
	CY4R	CGGTATGATAAGCCAGGGAAGT	
*inui*	PinF2	CGTATCGACTTTGTGGCATTTTTCTAC	60
	INAR3	GCAATCTAAGAGTTTTAACTCCTC	
*fieldi*	PfldF1	GGTCTTTTTTTTGCTTCGGTAATTA	66
	PfldR2	AGGCACTGAAGGAAGCAATCTAAGAGTTTC	

The PCR products were visualized using the QIAxcel Advanced System (a capillary electrophoresis system which enables fast size-based separation of nucleic acids (QIAGEN, Hilden, Germany) [29] and, as a potential alternative or for confirmation, standard agarose gel electrophoresis stained with GelRed™ (Biotium, Hayward, USA). The QIAxcel system automates the separation and analysis of nucleic acids based on size and charge, similar to traditional gel electrophoresis but with higher resolution, speed, and reproducibility. In this study, gel electrophoresis served as a supplementary method to visualize and analyse the PCR products, particularly useful for qualitative assessment of band size and presence, and as a backup in case of QIAxcel system malfunction.

**Fig 1:**
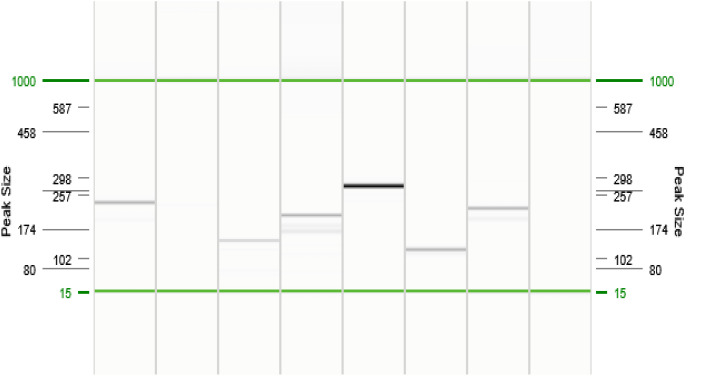
Nested PCR amplification products (Human malaria species). Gel representation of QIAxcel (QIAGEN, Hilden, Germany) capillary electrophoresis second amplification. Lines 1 = *Plasmodium* sp, Line 2 = Negative sample, Line 3 = *P. malariae*, Line 4 = *P. falciparum*, Line 5 = *P. knowlesi*, Line 6 = *P. vivax*, Line 7 = *P. ovale*, Line 8 = Negative sample.

**Fig 2:**
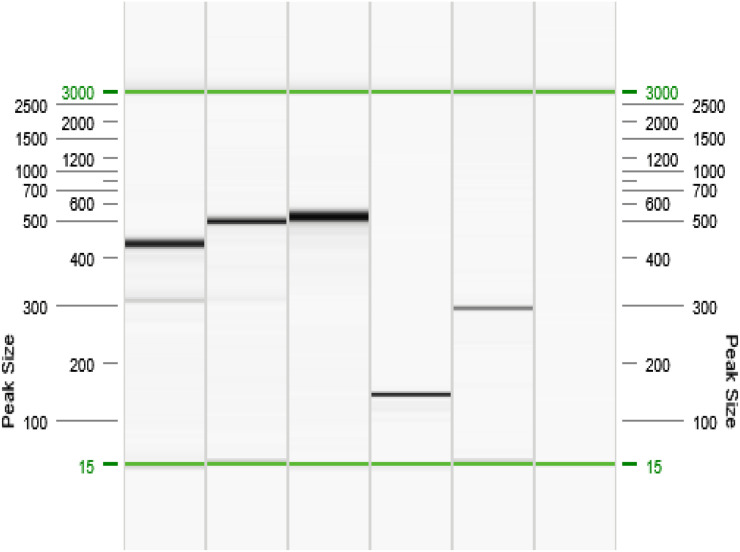
Nested PCR amplification products (Simian malaria species). Gel representation of QIAxcel (QIAGEN, Hilden, Germany) capillary electrophoresis second amplification. Lines 1 = *P. fieldi,* Line 2 = *P. inui*, Line 3 = *P. coatneyi*, Line 4 = *P. cynomolgi*, Line 5 = *P. knowlesi*, Line 6 = Negative sample.

Malaria species were identified based on specific fragment sizes: *P. falciparum* (206 bp), *P. vivax* (121 bp), *P. malariae* (145 bp), *P. ovale* (226 bp), and *P. knowlesi* (389 bp) for human malaria species (Fig 1); and *P. coatneyi* (504 bp), *P. cynomolgi* (137 bp), *P. fieldi* (421 bp), and *P. inui* (479 bp) for simian malaria species (Fig 2). Positive PCR products were subsequently sent to Nuclix Biosolution Sdn. Bhd. (Kuala Lumpur, Malaysia) for direct DNA sequencing to confirm species identities. A subset of PCR products (especially those indicating simian malaria) was purified and sequenced for quality assurance. The resulting sequences were compared to reference sequences in GenBank using BLAST to confirm species identity.

### Data analysis

All data were double-entered and validated in Microsoft Excel, then analyzed using R (RStudio IDE) and SPSS software. Descriptive statistics were used to summarize participant characteristics and infection rates. The prevalence of low-density malaria was calculated as the proportion of nPCR-positive individuals among those tested. Bivariate analyses (Chi-square or Fisher’s exact tests) were performed to explore associations between nPCR-confirmed infections and sociodemographic factors (age group, sex, occupation, and history of malaria). A p-value <0.05 was considered statistically significant. Due to the relatively low number of positive cases (n = 62), a comprehensive multivariate analysis could not be performed. However, notable trends from bivariate analyses were observed and are reported. The geospatial distribution of cases was mapped to identify the clustering of infections.

## Results

### Low-density infection prevalence and species composition

Out of 3,322 asymptomatic participants screened, none were positive by microscopy. In contrast, nPCR detected *Plasmodium* DNA in 62 individuals, yielding an overall low-density malaria prevalence of 1.86% (62/3,322; 95% CI: 1.46–2.39%). The prevalence was 6.2% (51/819; 95% CI: 4.77–8.10%) in Kota Marudu, 3.13% (3/96; 95% CI: 1.07–8.79%) in Keningau, and 0.46% (8/1,748; 95% CI: 0.23–0.90%) in RPS Pos Kemar. The majority were *P. malariae* (25 cases, 40.3% of all positives) and *P. vivax* (18 cases, 29.0%). *P. knowlesi*, the zoonotic species, accounted for 15 cases (24.2%). In addition, several infections with *P. falciparum* (1 case, 1.6%) and *P. cynomolgi* (1 case, 1.6%) were identified. There were two instances (3.2%) of mixed-species infection (each co-infection with *P. vivax* and *P. knowlesi*). Both of these mixed infections originated from the same village within RPS Pos Kemar in Perak. No *P. ovale*, *P. inui*, *P. coatneyi* or *P. fieldi* infections were detected.

### Geographic infection distribution

**Fig 3:**
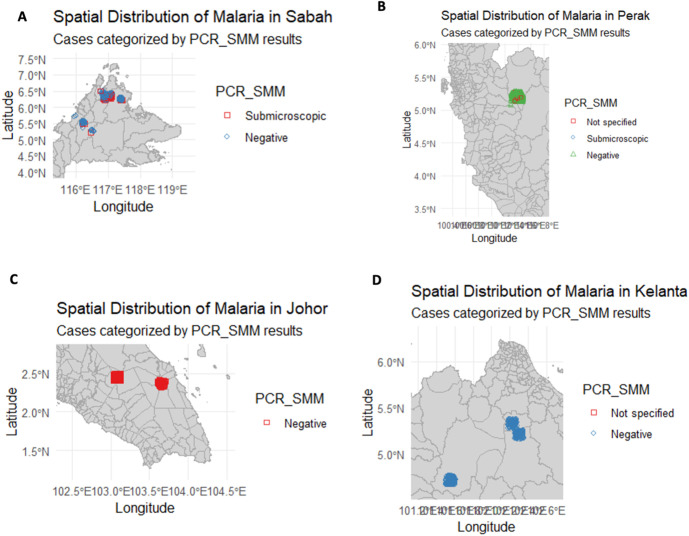
Spatial distribution of low-density malaria in (A) Sabah, (B) Perak, (C) Johor and (D) Kelantan (This map was generated by the authors using R spatial packages and RStudio IDE. It is published under a CC BY 4.0 license).

All 62 nPCR-positive individuals were found in three localities: two villages in Sabah (Kota Marudu: 51 cases; Keningau: 3 cases) and one community in Hulu Perak, Perak (Pos Kemar resettlement: 8 cases). The prevalence of low-density malaria was 6.2% (51/819) in Kota Marudu, 3.13% (3/96) in Keningau, and 0.46% (8/1,748) in RPS Pos Kemar. By contrast, no positive case was detected among participants from the surveyed localities in Johor (Segamat and Mersing, 0/239 combined) or Kelantan (Gua Musang and Kuala Krai, 0/422) (Table 2). Fig 3 illustrates the locations of the positive cases.

**Table 2:** Prevalence and Species Distribution of Submicroscopic Malaria Detected by nPCR in Selected Localities with High RV Index in Malaysia (n = 3,320).

State	District	Number of samples tested	Human and simian malaria species										Number of samples positive	Number of samples negative
			*Pm*	*Pv*	*Pf*	*Po*	*Pk*	*Pcy*	*Pin*	*Pct*	*Pfd*	Mixed infection		
Sabah	Kota Marudu	817	24	13	0	0	14	0	0	0	0	0	51	766
	Keningau	96	0	0	1	0	1	1	0	0	0	0	3	93
Perak	RPS Pos Kemar	1748	1	5	0	0	0	0	0	0	0	2	8	1709
Johor	Segamat	186	0	0	0	0	0	0	0	0	0	0	0	178
	Mersing	53	0	0	0	0	0	0	0	0	0	0	0	53
Kelantan	Gua Musang	207	0	0	0	0	0	0	0	0	0	0	0	207
	Kuala Krai	215	0	0	0	0	0	0	0	0	0	0	0	215
Overall prevalence		3322	25	18	1	0	15	1	0	0	0	2	62	3221

*Pm = P. malariae; Pv = P. vivax; Pf = P. falciparum; Po = P. ovale; Pk = P. knowlesi; Pcy = P. cynomolgi; P. in = P. inui; Pfd = P. fieldi.*

### Characteristics of infected individuals

We compared the demographic and risk profiles of individuals with low-density infections (n = 62) to the study population (n = 3,322). The age distribution of infected individuals skewed toward older age groups: 67.7% (42/62) were adults aged ≥17, 30.6% (19/62) were school-aged children 6–16 years and only 16.1% (10/62) were young children under 6 years (values adjusted). It contrasts with the age composition of the study population (33.9% were 6–16 years and 10.0% were <6 years) (Table 3). However, the difference in infection rate by age group did not reach statistical significance (χ² test, p = 0.08). There was no significant difference in low-density infection prevalence between females and males (prevalence 1.64% vs 2.10%, p = 0.52). Similarly, having a history of malaria (self-reported past infection) was not significantly associated with low-density infection (positive rate 2.85% in those with prior malaria vs 1.81% in those with no history, p = 0.47).

**Table 3:** Demographic characteristics of study participants and distribution by infection status and *Plasmodium* species.

Characteristics	Number of Participants (Column %)	Sabah		Perak	Kelantan		Johor	
		Kota Marudu	Keningau	RPS Pos Kemar	Kuala Krai	Gua Musang	Mersing	Segamat
Total participants	3322	817	96	1748	215	207	53	186
**Age Group**								
0–5 y/o (pre-school children)	334	86	0	195	7	27	6	13
6–16 y/o (School children)	1121	281	6	643	58	73	19	41
17 ≥ , (household income earners/housewives)	1830	432	90	902	149	106	28	123
Missing	37	18	0	8	1	1	0	9
**Gender**								
Female	1711	461	42	906	91	101	24	86
Male	1579	339	54	835	124	106	29	92
Missing	32	17	0	7	0	0	0	8
**Marital Status**								
Married	1367	355	66	627	119	72	25	103
Single	1836	426	29	1073	88	128	26	66
Divorced/Widowed	57	8	1	33	6	6	2	1
Missing	62	28	0	15	2	1	0	16
**Ethnicity**								
Indigenous Sabah (Bumiputera)	873	780	93	0	0	0	0	0
Orang Asli	2242	0	0	1736	71	204	53	178
Malay	147	2	0	2	143	0	0	0
Chinese	1	0	1	0	0	0	0	0
Other Communities	17	13	2	2	0	0	0	0
Missing	42	22	0	8	1	3	0	8
**History of Malaria**								
Yes	281	74	41	148	12	3	0	3
No	2989	708	55	1591	203	204	53	175
Missing	52	35	0	9	0	0	0	8
**Occupation**								
Agricultural and Natural Resources Workers	391	90	5	208	35	0	3	0
Service and Education Sector	136	44	16	26	40	7	0	3
Self-Employed and Business Owners	367	72	1	138	17	38	9	92
Students and Non-Working Individuals	1674	386	12	1003	82	117	26	48
Transport and Labour Workers	5	1	0	4	0	0	0	0
Community Leaders and Religious Figures	7	2	0	5	0	0	0	0
Housewives and Caregivers	660	170	12	351	40	12	15	28
Missing	82	52	50	13	1	33	0	15
**PCR-Confirmed Malaria Species:**								
*P. malariae*	25	24	0	1	0	0	0	0
*P. vivax*	18	13	0	5	0	0	0	0
*P. falciparum*	1	0	1	0	0	0	0	0
*P. knowlesi*	15	14	1	0	0	0	0	0
*P. cynomolgi*	1	0	1	0	0	0	0	0
*Pv + Pk*	2	0	0	2	0	0	0	0
Negative	3221	766	93	1709	215	207	53	178
Missing	39	0	0	31	0	0	0	8

Regarding ethnicity and community, the most positive cases (93.5%) occurred in indigenous communities: 38 cases were among Dusun and other indigenous peoples of Sabah, and eight were among Orang Asli in Hulu Perak. Only one case was detected in a person of Malay ethnicity in Keningau, Sabah, and none in non-indigenous residents of other areas. In Kota Marudu, most positives were among adults engaged in agriculture or forest gathering. At Pos Kemar, Hulu Perak, they lived in a forest-fringe environment. Meanwhile, in Johor and Kelantan, they primarily lived in less forested settings or had livelihoods less tied to forest exposure, such as villages near towns.

## Discussion

### Continued malaria transmission in an elimination setting

Our findings reveal that malaria transmission persists at a subclinical level in certain parts of Malaysia despite the absence of microscopically confirmed cases. In two districts of Sabah and one locality in Peninsular Malaysia (Perak), we detected low-density *Plasmodium* infections in individuals who appeared healthy and would not have been identified through routine surveillance. It underscores that achieving zero reported cases may not necessarily equate to truly interrupting transmission. Malaysia’s overall malaria burden has declined dramatically over the past decade, for example, in 2018, approximately 4,630 malaria cases were reported nationwide, of which ~89% were due to *P. knowlesi* and only 11% were imported or introduced human malaria cases [3,4,16,30,31]. These statistics illustrate that indigenous human malaria was nearly eliminated, yet *zoonotic* malaria remained prevalent. In this context, our study provides molecular evidence of residual malaria parasites circulating among community members in supposedly malaria-free zones. The detection of *P. knowlesi* DNA in asymptomatic individuals stands out as a significant observation. Although *P. knowlesi* often causes acute febrile illness, our results show it can also exist at low densities without symptoms, potentially serving as a silent bridge between the monkey reservoir and the human population [32].

### Hidden reservoirs and underestimation of prevalence

The use of sensitive nPCR in this study revealed malaria prevalence of 1.86%, which was entirely missed by microscopy. This finding confirms that reliance solely on microscopy underestimates true infection rates in low-endemicity settings [33]. Even though 1.86% seems like a low percentage, in absolute terms, 62 infections would have gone undetected and untreated. These individuals were not treated for malaria due to several reasons: (1) the study protocol required non-intervention for PCR-positive, microscopy-negative individuals; (2) they were asymptomatic; and (3) national malaria treatment guidelines, aligned with the WHO Guidelines for the Treatment of Malaria, which recommends antimalarial therapy only for individuals who test positive by microscopy or present with clinical symptoms [2,18].

Notably, all submicroscopic infections in this study were low-density and undetectable by microscopy, confirming that conventional diagnostic methods would have classified these communities as malaria-free. This underscores the blind spots in standard surveillance systems and highlights the urgent need to integrate more sensitive molecular diagnostics into elimination settings. While asymptomatic, these low-density carriers can still infect mosquitoes and sustain transmission within the community [34]. Our results align with observations from other regions transitioning to elimination: a substantial proportion of infections can be sub patent and thus evade standard diagnostics [35,36]. For instance, studies in Southeast Asia and other low-transmission settings have reported that 20–50% of *Plasmodium* infections are only detectable by PCR or other molecular methods [34,37]. By confirming the existence of such a hidden reservoir in Malaysia, our study highlights a critical vulnerability in the current surveillance system. It also validates the strategic shift towards including more sensitive diagnostic tools (like PCR or highly sensitive rapid tests) in malaria elimination programs. Without addressing low-density infections, areas that are declared malaria-free may still harbour parasites that could reinitiate transmission if conditions become favourable, e.g., reintroduction of vectors or the arrival of susceptible individuals) [31,38–41].

This situation highlights a critical gap in malaria case management during the elimination phase. Low-density carriers, while asymptomatic, can still infect mosquitoes and perpetuate local transmission [35,42–44]. As such, untreated reservoirs of infection like those identified in our study, pose a threat to sustaining elimination gains [45]. To address this, national policies may need to be revised to include molecular diagnosis as a trigger for treatment in specific contexts, particularly in high-receptivity or outbreak-prone areas [18,46]. Treating confirmed submicroscopic carriers could become an important strategy in the final stages of malaria elimination, where every undetected infection represents a potential source of resurgence [15,35,47].

The species composition of low-density infections in our study sheds light on transmission dynamics. We found *P. malariae* and *P. vivax* as the most common species, followed by *P. knowlesi*. The near-absence of *P. falciparum*, historically a major cause of malaria in Malaysia, points to the success of national control efforts and potentially to the species’ lower likelihood of persisting at low densities in asymptomatic carriers when compared to *P. vivax* or *P. malariae*. *P. vivax* is well-known for its ability to form dormant liver stages (hypnozoites) and cause relapses, leading to low-grade parasitaemia that microscopy might miss. *P. malariae* is characterized by chronic low parasitaemia infections lasting for years. Therefore, the prominence of these two species among submicroscopic cases is unsurprising and consistent with their biological traits.

Meanwhile, the detection of asymptomatic *P. knowlesi* infections is noteworthy. It suggests that some zoonotic infections, likely from mosquito vectors feeding on macaques, may not always produce acute symptoms, and thus may evade clinical detection. These silent *P. knowlesi* carriers could represent an under-recognized transmission route, complicating zoonotic malaria control efforts and emphasizing the need for One Health-based strategies.

### Focal transmission and environmental factors

One aspect of our results is the focal nature of low-density malaria transmission. The infections were not randomly scattered across all high-risk areas but clustered in specific communities. characterized by hilly, forested terrain and rural indigenous settlements. Similarly, Pos Kemar in Hulu Perak is a forest-fringe Orang Asli resettlement area in Peninsular Malaysia. These areas share environmental and ecological features conducive to malaria transmission, including dense forest cover that supports populations of *Anopheles* mosquitoes (particularly the *leucosphyrus* group), the presence of macaque monkeys, the natural hosts of *Plasmodium knowlesi,* and human communities living or working in close proximity to forested ecosystems. By contrast, the localities surveyed in Johor and Kelantan (which showed zero submicroscopic cases) have relatively more developed landscapes or fewer interface opportunities between people, vectors, and wildlife, e.g., villages located farther from deep forests or with more intensive vector control measures in place [16,30,48], suggests that environmental factors play a significant role in residual malaria transmission.

The communities in Sabah where positive cases were detected are predominantly remote villages where residents rely on subsistence farming and frequently enter forested areas for farming or foraging. Such activities likely increase exposure to *Anopheles* mosquito vectors and potentially to simian malaria reservoirs. Similarly, the Orang Asli population at Pos Kemar in Hulu Perak lives in a forest-fringe environment with frequent human–macaque contact. Occupationally, most infected individuals were farmers or plantation workers, which is consistent with greater exposure to mosquito bites due to outdoor and nighttime activity. While the sample size of infected individuals in our study was limited, these patterns support the hypothesis that environmental and occupational risk factors contribute to low-density malaria transmission.

Our study supports the idea that even within a country, “hotspots” of lingering transmission can exist due to localized conditions. Such hotspots demand targeted attention. For example, the indigenous communities in Sabah with ongoing low-density malaria may benefit from focused interventions like enhanced vector control at forest fringes, community education on personal protection, and perhaps targeted mass screen-and-treat campaigns using ultrasensitive tests. In Hulu Perak, entomological surveillance could be intensified to monitor *Anopheles* populations, and interventions like insecticide-treated hammocks or nets for Orang Asli who go into the forest might be warranted [49,50]. Incorporating geospatial analysis (as we have done to map cases) helps identify environmental risk factors such as proximity to water bodies (mosquito breeding sites) or monkey habitats.

### Host factors and immunity

The demographic patterns observed provide insights into host factors that might influence low-density infections. We found a higher proportion of adults among the infected people, which could be explained by acquired immunity. This is in contrast with the overall age distribution of the study population, where 33.9% were aged 6–16 years and 10.0% were under 6 years, suggesting that adults constituted a disproportionately high share of infections. In areas with historically low or intermittent transmission, adults may have had just enough exposure over their lifetime to develop partial immunity that allows them to carry low levels of parasites without symptoms, whereas children (with less exposure) might either not sustain infections as long or would develop symptoms at lower parasite densities and get treated [51]. It is a plausible explanation for why more adults were PCR-positive while apparently remaining healthy. Similar age-related trends have been noted in other studies, where subclinical malaria infection rates tend to peak in older age groups once clinical malaria declines in a community [39,52]. However, our data did not show a statistically significant difference by age or gender, likely due to the limited number of positive cases. The lack of significant association with gender suggests that both men and women were exposed relatively equally in these communities. However, their exposure routes might differ, men often work in forests, and women in farms or around the village. We also found no clear link with prior malaria history, indicating that having had malaria before did not predispose or protect individuals from carrying low-density infection. It could mean that infections are more tied to recent exposure opportunities than long-term immunity or that any immunity gained is strain/species-specific or short-lived. Mild malaria symptoms may be unrecognized by individuals; thus, some people with a prior malaria history may not have reported being infected before. Therefore, perhaps testing for *anti-Plasmodium* antibodies would be a better way to determine a prior malaria history [53,54].

These observations highlight that human factors and immunity modulate low-density malaria prevalence. People who frequently enter vector-rich environments (regardless of gender) are at risk of acquiring new infections. However, those with some immunities (often adults in endemic areas) are more likely to harbour infections at low-density levels. It reinforces the need for community engagement: educating all members (men, women, farmers, loggers and others) about the risk of asymptomatic malaria and encouraging preventive measures such as consistent use of bed net and mosquito repellents and avoiding peak mosquito hours [42,55].

### Implications for malaria elimination efforts

From a public health perspective, identifying low-density malaria foci in Malaysia has several important implications. First, malaria elimination programs must combine molecular surveillance and conventional methods, especially in areas with zero cases by standard reporting [56,57]. Periodic mass blood surveys or targeted screening of high-risk groups using PCR or new highly sensitive rapid tests (if available) could be invaluable in the early detection of residual transmission. In our study, if PCR screening had not been done, these communities would be deemed malaria-free with a false sense of security. As Malaysia approaches the goal of eliminating human malaria, integrating such active case detection strategies will be crucial to catching what the routine surveillance misses [31,38,58].

Second, tailored intervention strategies are required to address residual pockets of malaria. Blanket approaches used during high malaria transmission may no longer be cost-effective; resources should be directed to known hotspots. In Sabah and Hulu Perak, for example, health authorities might intensify monitoring of forest-exposed populations and provide prophylactic measures or test-at-entry for people moving between endemic and non-endemic areas. *P. vivax* and *P. malariae* infections detected only by PCR still harbour transmission potential; thus, radical cure of *P. vivax*, with primaquine or tafenoquine to clear hypnozoites, could be considered even for asymptomatic carriers to prevent relapse and further transmission. Likewise, detecting *P. knowlesi* in humans calls for a One Health approach: collaboration with wildlife and vector specialists to understand and reduce the spillover from macaques to humans. It could involve vector control strategies targeting the zoophilic vectors in forest areas or strategies to minimize human-macaque interaction in hotspot regions [2,44,59–61].

Third, our findings emphasize the importance of sustained vigilance. As nations approach elimination, there may be a tendency to scale back malaria control resources and investments. However, our findings demonstrate the risks of reducing vigilance, as hidden infections can persist unnoticed and potentially cause resurgence if surveillance efforts are relaxed. Malaysia’s example will resonate with other countries in the E-2020 initiative and beyond – the endgame of elimination requires as much rigour as the earlier stages, if not more. Surveillance systems must be sensitive and responsive; for instance, any PCR-positive detection should trigger an investigation and a localized response, such as focal screening and vector assessment, treating it almost like an index case of an outbreak [47,62,63].

Finally, our results have significant implications for public health policy. Detecting persistent low-density infections in areas declared malaria-free highlights the limitations of relying exclusively on symptomatic case detection and traditional microscopy-based surveillance. These undetected infections present hidden reservoirs that, if left unaddressed, could severely undermine elimination achievements. Consequently, national malaria control programs should consider revising current surveillance and treatment guidelines, incorporating molecular diagnostics or highly sensitive field diagnostic tools, such as loop-mediated isothermal amplification (LAMP) or ultra-sensitive rapid diagnostic tests (uRDTs). Proactively identifying and treating asymptomatic carriers would substantially reduce transmission potential, particularly in high-receptivity areas, ultimately bridging critical gaps toward achieving lasting malaria elimination [64–66].

### Limitations and future research

This study had several limitations. First, the cross-sectional design provides a snapshot in time; we may have missed seasonal variations in low-density infection. Conducting surveys in different seasons (e.g., after the rainy season when vector densities peak) might detect additional infections. Second, the overall number of positive cases was relatively low (n = 62). This limited the statistical power to detect moderate associations between risk factors and infection status and, crucially, restricted our ability to perform comprehensive multivariate analyses. Such analyses could have provided a deeper examination of confounding factors and independent predictors associated with low-density infections, beyond the bivariate associations explored. A larger sample size or pooling data from multiple surveys could help in firmly identifying risk factors for carrying low-density malaria and enable more robust statistical modelling. Third, it is important to acknowledge that PCR detects parasite nucleic acids (DNA), and thus does not differentiate between viable and non-viable parasites. While our findings indicate the presence of *Plasmodium* DNA, the detection of non-viable parasites could potentially overestimate the true prevalence of active infections capable of onward transmission. This limitation should be considered when assessing the transmission potential inferred from PCR positivity.

The socio-demographic data collected via the questionnaire were crucial for understanding factors influencing malaria prevalence. However, our study lacked detailed data on malaria-related knowledge among participants, such as transmission routes, prevention methods, previous malaria infections, and antimalarial medication use, limiting our ability to correlate clinical manifestations or outcomes with low-density infections. Future research should address these gaps to provide comprehensive insights into community-level factors influencing malaria transmission and persistence. Incorporating these data could better inform targeted education, intervention programs, and clinical management strategies, potentially reducing residual transmission in high-receptivity and vulnerability (RV) areas.

Another limitation is that we did not directly assess vectors or animal reservoirs as part of this study. Incorporating an entomological survey (e.g., capturing mosquitoes in and around the communities to test them for *Plasmodium* infection) and a survey of local macaque populations for *P. knowlesi* could provide a more complete picture of the transmission cycle. Future research should adopt a One Health perspective, examining the interplay between human low-density malaria carriers, mosquito vectors, and macaque hosts. Future research should also examine these environmental and ecological determinants more closely, as understanding them can inform tailored vector control strategies. For instance, if particular landscape features correlate with low-density malaria case clusters, those areas can be prioritized for interventions like focal spraying or deployment of mosquito traps. Longitudinal studies following individuals over time would also be valuable to see how long low-density infections persist, and whether they eventually flare into detectable parasitaemia or get cleared. Understanding the natural history of these infections can inform if and when intervention is needed (for instance, treating asymptomatic carriers pre-emptively).

Finally, there is a need to explore and validate field-friendly diagnostic tools that can approach the sensitivity of PCR. Techniques such as loop-mediated isothermal amplification (LAMP) [65,67,68] or newer ultra-sensitive rapid tests (uRDTs) [64,66,69] for *Plasmodium* antigens have shown promise. We support further evaluation of these tools in community settings like ours. If health workers could have a kit to detect low-density malaria on-site, it would revolutionize elimination surveillance. Operational research to integrate such tools into routine programs should be prioritized. It would also be helpful to investigate whether environmental interventions, such as targeted vector control mentioned above, can reduce residual infections.

## Conclusion

In conclusion, our study demonstrates that low-density malaria infections persist in selected malaria-free declared areas of Malaysia, posing a hidden threat to malaria elimination. While traditional surveillance indicates success in interrupting transmission, molecular evidence reveals that a low-level parasite reservoir persists among asymptomatic individuals. Addressing this silent reservoir is essential for Malaysia to achieve and sustain a genuinely malaria-free status. It will require enhanced surveillance with more sensitive diagnostics to identify and treat even asymptomatic, low-density infections. It will require strong community engagement and education, ensuring that at-risk populations remain vigilant and cooperative with elimination efforts even without clinical cases.

Targeted intervention strategies should be implemented in the identified hotspots, for instance, intensified vector control in and around the affected communities, proactive screening of high-risk groups, and a One Health approach to managing zoonotic malaria sources. By tailoring measures to the local context (considering human behaviour, vector ecology, and environmental factors), Malaysia can mitigate the risk of malaria reintroduction.

The experience from this study offers a valuable guidance for other countries nearing elimination: hidden pockets of malaria can exist undetected and must be proactively sought out. As global malaria elimination efforts progress, strategies must evolve to “find the needle in the haystack.” Malaysia’s commitment to elimination, which is aligned with global targets, will benefit from incorporating these findings. With sustained vigilance, innovative tools, and targeted action, the goal of a malaria-free Malaysia is within reach, and the hard-won gains in malaria control can be safeguarded for the long term.
